# Supplementary material for: Passive optical time-of-flight for non line-of-sight localization
Source: Nat Commun. 2019 Jul 26;10:3343. doi: 10.1038/s41467-019-11279-6 (PMC6659653; doi:10.1038/s41467-019-11279-6)
Supplement: Supplementary file 1 — Supplementary information [file 41467_2019_11279_MOESM1_ESM.pdf]

Passive optical time-of-flight for non line-of-sight localization
Supplementary Information

Jeremy Boger-Lombard[1] and Ori Katz[1*]

[1]Applied Physics Department, The Hebrew University of Jerusalem,
Jerusalem, 9190401, Israel

*orik@mail.huji.ac.il

Supplementary Note 1:

Optical setup

The full experimental setup for imaging through a diffusive barrier is given in Supplementary Figure 1. The light source used for the experiments was a white-light LED source (MWWHF1, Thorlabs), which was split into four by coupling to a fiber bundle (BF42HS01 Thorlabs). The sources in all the experiments were 2-3 tips of the four fiber bundle ends. The sources were positioned behind a highly scattering diffuser (light shaping diffuser, $80°$ scattering angle, Newport).

On the other side of the diffusive barrier, a 4-f telescope ($f_1 = 150$mm, $f_2 = 180$mm) imaged the barrier's surface on a movable mask, comprised of two small apertures (for 2D localization), or two pairs of apertures, for 3D localization. The mask was placed at the front focal plane of a lens ($f_3 = 100$mm). A cooled sCMOS camera (Andor Neo 5.5) placed at the back focal plane of the lens, records the diffraction pattern of the scattered light that is transmitted through the double aperture mask.

From the ratio of the focal lengths of the 4-f telescope, the dimensions and separation of the apertures on the mask are reduced by a factor of 1.2 when imaged on the diffuser.

Circular apertures were used in our mask-based experiments, and rectangular apertures were used only in the SLM-based experiments, due to the square dimensions of the SLM pixels.

For the experiments with reflective objects (Fig. 5), the diffuser used was Newport light shaping diffuser with a scattering angle of $40°$.

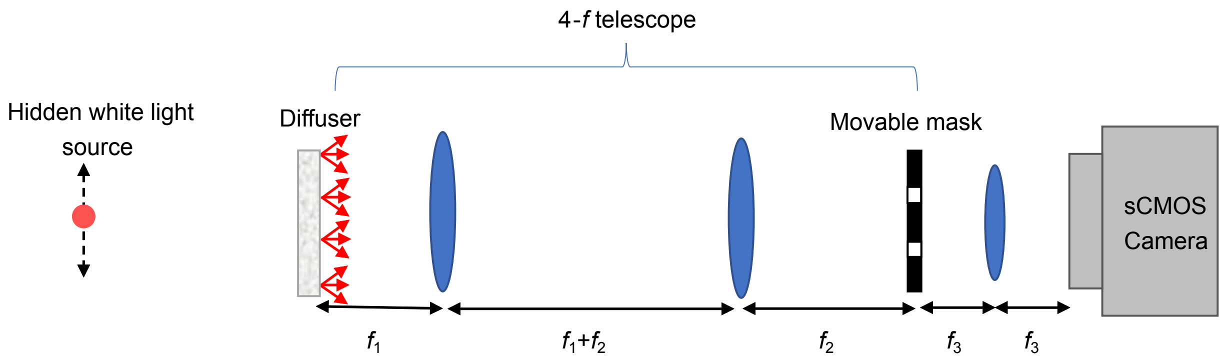

Supplementary Figure 1: **Setup for passive TOF through a highly scattering medium:** a white-light LED point source is hidden behind a diffusive barrier. A double aperture mask that is 4-f imaged on the barrier, selects diffused light from two points on the barrier to be interfered on an sCMOS camera. The mask is translated vertically to measure different points on the barrier.

Supplementary Note 2:

Results using a dynamic programmable mask

In order to go beyond the limitations of a static double-aperture mask that is mechanically scanned across the barrier, the setup shown in Supplementary Figure 2a was constructed. In this system, the double aperture mask is replaced by a programmable digital amplitude mask constructed using a spatial light modulator (SLM). For amplitude shaping, the phase-only SLM (Holoeye Pluto BB) was placed between two linear polarizers in a cross-polarization configuration.

The setup for a dynamic programmable mask is conceptually identical to the setup using a mechanically-scanned fix mask (Supplementary Figure 1), with two main technical differences: The first is that in order to obtain a high contrast amplitude mask using the specific liquid crystal SLM model (Holoeye PLUTO BB), which had significant chromatic dispersion, a narrow band-pass filter (BPF) with a 10nm bandwidth (FB550-10 Thoralbs) was placed before the camera. This reduces the light utilization efficiency of this setup, and could be improved by using less dispersive SLMs, amplitude only SLMs, or potentially MEMS based SLMs. The second difference of the programmable mask setup of Supplementary Figure 2a, was that the reflective SLM was placed at a close distance (< 5cm) to the diffusive barrier, instead of being 4-f imaged on it. Even with such imperfect conjugation, two light sources could be simultaneously separated and localized with our approach through a diffusive barrier, by displaying a double aperture mask at different positions on the SLM (Supplementary Figure 2b,c). In this experiment the diffusive barrier was a light shaping diffuser, with a scattering angle of $5°$ (Newport), and the mask was generated using the SLM to produce rectangular reflective slits 0.24mm wide with a separation

of 3.2mm .

Supplementary Figure 2b presents the results of fringe localization from 18 different positions of the displayed double mask aperture, achieved without any mechanical scanning: each row in Supplementary Figure 2b, is the fringe envelope amplitude at different positions of the camera, extracted using spectrogram analysis (see Supplementary Figure 3). The reconstructed sources positions from these measurements are shown in Supplementary Figure 2c.

A programmable mask is advantageous over a mechanically scanned fixed mask as it does not require mechanical scanning, it can straightforward implement advanced multiplexing approaches that may be used for reconstructing the scene and the source image from a single camera exposure, as done in aperture masking interferometry in astronomy[1].

The main disadvantage of a programmable mask is the lower contrast and transmission compared to a mechanical mask, and potential chromatic aberrations. In our implementation the chromatic aberrations required the use of a narrow bandpass filter, which not only resulted in lower light utilization efficiency, but also reduced the temporal resolution by an order of magnitude, to be of the order of 100fs, still considerably better than the response time of the state of the art detectors. The lower temporal resolution can be observed in a larger width of the curves shown in Supplementary Figure 2b.

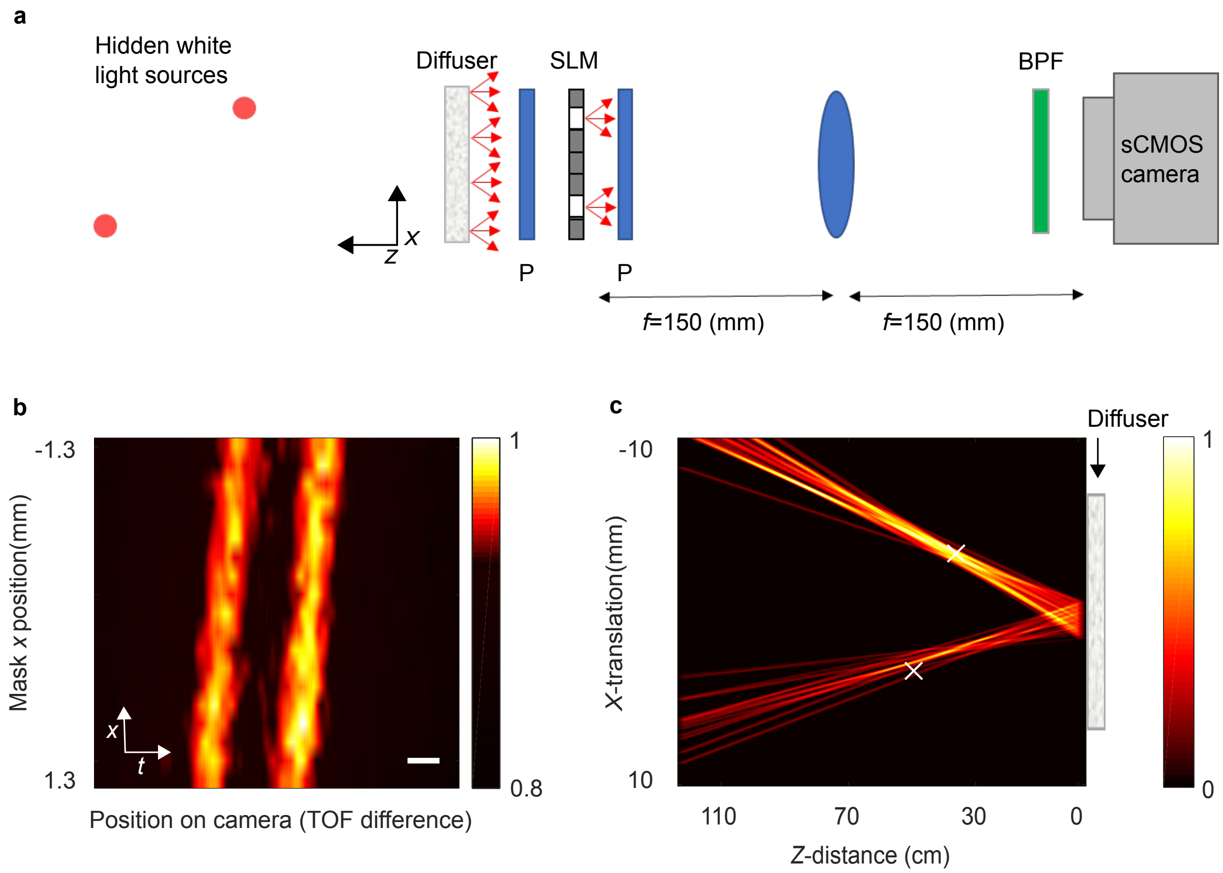

Supplementary Figure 2: **Localization of multiple hidden sources using a dynamic programmable mask.** **(a)** Setup: two white light LEDs are hidden behind a diffusive barrier. The light is collected from two points on the barrier by displaying a double aperture amplitude mask on an SLM. The interference pattern is recorded on a camera (Zyla 4.2 plus). **(b)** Interference fringes envelope as a function of the position of the double aperture mask, shifted without moving elements. The fringes provide the TOF information.**(c)** Scene reconstructed from (b) by back-projection. The real positions are marked by 'x'. Scale bar: 100fs.

Supplementary Note 3:

Constructing the spatio-temporal TOF (x,t) maps

Supplementary Figure 3a shows a raw camera image taken with the setup of Supplementary Figure 1 . The image seems to be a random, low contrast, speckle pattern, as would be seen through a highly scattering barrier. However, as a result of the double aperture mask, low coherence (white-light) fringes are present in the image, at a position which reflects the TOF

difference (Supplementary Figure 3a, red arrow). The fringes are localized around the zero delay time providing the optical path difference of the light from the hidden light source to the two apertures.

In order to allow detection of the fringes, we made sure that the fringes period is considerably smaller than the speckle grain size. This was ensured by using double aperture masks having an aperture separation distance that is larger than the size of each aperture. Thus, the fringes can be localized by high-pass (or bandpass) filtering the camera image, around the fringes spatial frequency. One possible approach to perform such filtering is via a 2D Hilbert transform, as shown in Fig. 2.

Another equivalent processing approach is to perform a spectrogram analysis for each camera row. The result of such an analysis is shown in Supplementary Figure 3b: The shown result is the average of spectrogram analysis performed over each of the camera image rows, after averaging each 10 camera rows. The spectrogram analysis performs a short time Fourier transform (STFT) around each horizontal pixel position with a chosen window length (here, 128 pixels). This provides spatial frequency information to be analyzed with a spatial resolution of the window length. The direct result of such simple spectrogram analysis shows a clear spectral peak that is localized around the position of the interference fringes. Taking the relevant spectrogram row (i.e. spatial frequency of the interference fringes, Supplementary Figure 3b, dashed box) provides the position of the fringes for this particular mask position. Repeating this process for different mask positions provides the spatio-temporal TOF trace of Supplementary Figure 3c, which allows the source localization (here a single source).

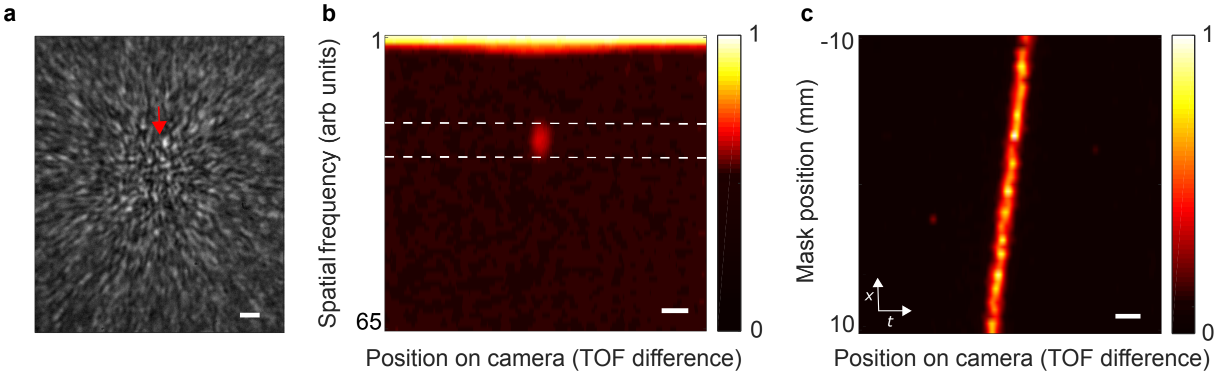

Supplementary Figure 3: **Extracting the fringes position using spectrogram analysis.(a)** Raw camera image (after median filtering).**(b)** Spectrogram of one camera row, averaged over all camera rows in a single camera image. The resulting amplitude peak, at the row corresponding to the fringes spatial-frequency, marks the interference fringes position on the camera, i.e. the measured TOF. **(c)** Repeating the process shown in (a-b) for different mask positions, and plotting the resulting spectrograms at the fringes' spatial frequency (row in (b) marked by a dashed box), provides the spatiotemporal TOF trace from which the scene can be reconstructed. Scale bars: 100fs.

Supplementary Note 4:

Localizing sources from hyperbolas intersection

Several approaches can be used in order to reconstruct the positions of the sources from the spatio-temporal TOF traces (e.g. Supplementary Figure 3c, and Supplementary Figure 4). Such approaches include filtered back-projection [2], or similar inversion procedures, tracing the light back to ellipsoids [3] or spheres [4]. In our approach, each fringes intensity peak in the spatio-temporal (x-t) trace (e.g. Supplementary Figure 3c) provides a TOF *difference*, and thus localizes the sources on a hyperbola.

To demonstrate a simple localization approach, we have implemented a back-projection procedure, which sums up the contributions of all the hyperboloids retrieved from each peak of the fringes detected intensity. To account for the experimental setup mounting inaccuracies, the fringe position to TOF delay was determined based on a set of calibration measurements with set of sources at known positions. Example results of such calibration measurements over a field of view of 80cm x 20mm (z,x) are shown in Supplementary Figure 4. For a coarse calibration, measurements for a set of 63 source positions (7 x 9, in (x,z) respectively) were taken, when each curve contains 40 different mask positions. For noise filtering and smoothing the results of the reconstructed hyperbolas were convoluted with a rectangular smoothing kernel. For the localization around a corner (Fig. 4), due to the roughness of the wall surface, the fringes peak positions varied considerably more as a function of the mask position (Fig. 4d), compared to the experiments with the diffusive barrier. In order to reduce the resulting errors in the measured TOF, the hyperboloids were reconstructed from the average fringes positions over 5 adjacent mask positions.

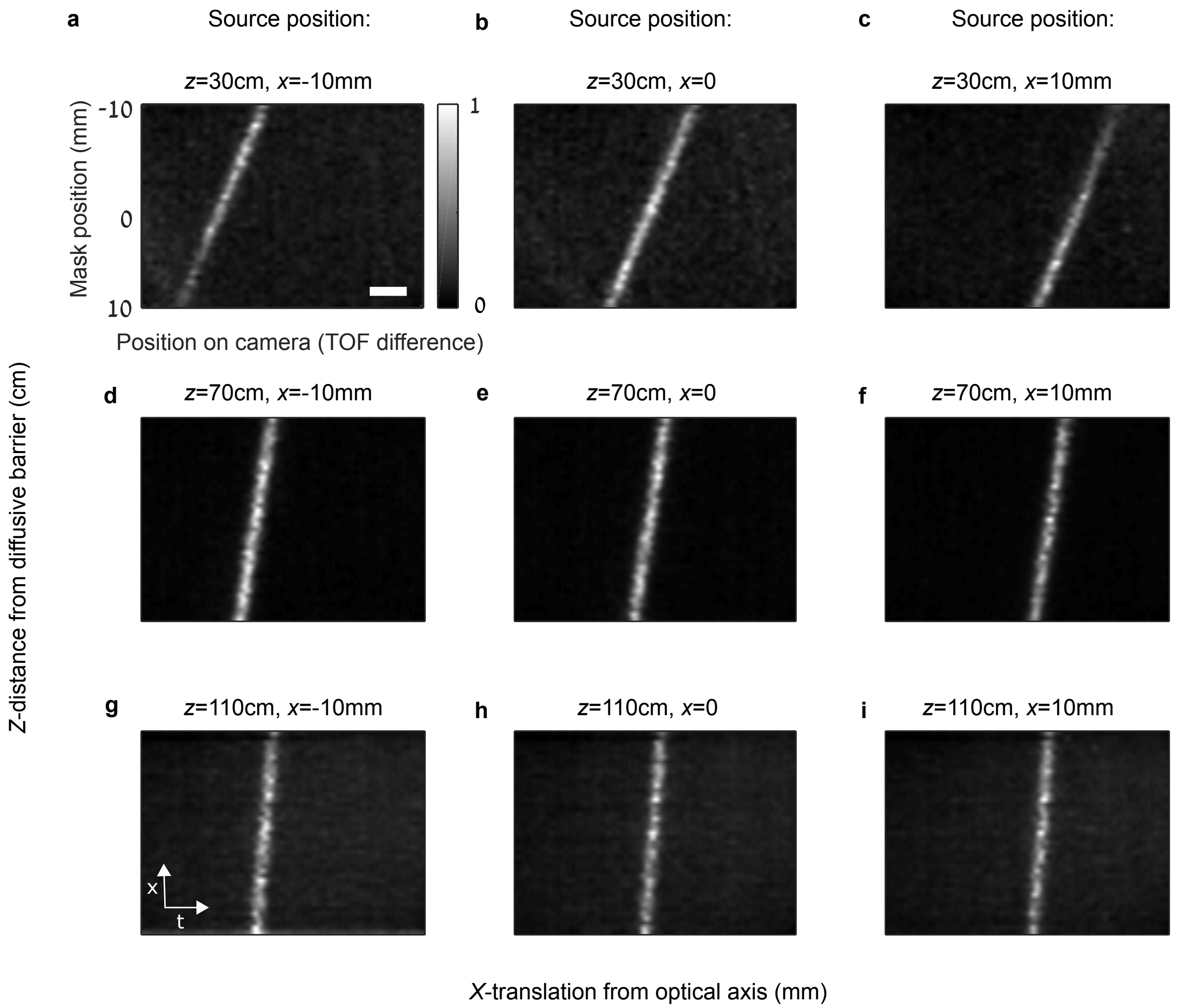

Supplementary Figure 4: **Spatio-temporal TOF traces for different source positions, used for system calibration and verification.** Nearby sources **(a-c)** show a spatiotemporal curve with a larger slope than far-away sources **(g-i)**. The traces horizontal center position reflects the transverse (x) position of the source. Scale bars: 100fs.

Supplementary Note 5:

TOF resolution and system geometry consideration

5.1. TOF resolution of the different sources used

The temporal TOF resolution of our approach is dictated by the source coherence time, i.e. the envelope of the source field autocorrelation. Which, according to the Wiener-Khinchin theorem, is given by the Fourier transform of the source power spectrum. In our experiments we have used two light sources: a white light LED and a tungsten-halogen lamp (Thorlabs OSL2). These two sources have different spectra (Supplementary Figures 5a,d), and thus the autocorrelation envelope of these two sources, as calculated by Fourier-transforming their spectra, have slightly different temporal widths (Supplementary Figures 5b,e). Specifically, the full width half maximum (FWHM) of the of the field auto-correlation envelope is $\tau_{FWHM} \approx 6.6$fs for the LED source , and $\tau_{FWHM} \approx 6.1$fs for the halogen lamp (Supplementary Figures 5b,e). These resolutions are three orders of magnitude better than ultrafast detectors used in conventional TOF techniques, which are of the order of 15ps using a streak camera [2], and 8ps using SPAD detectors[4].

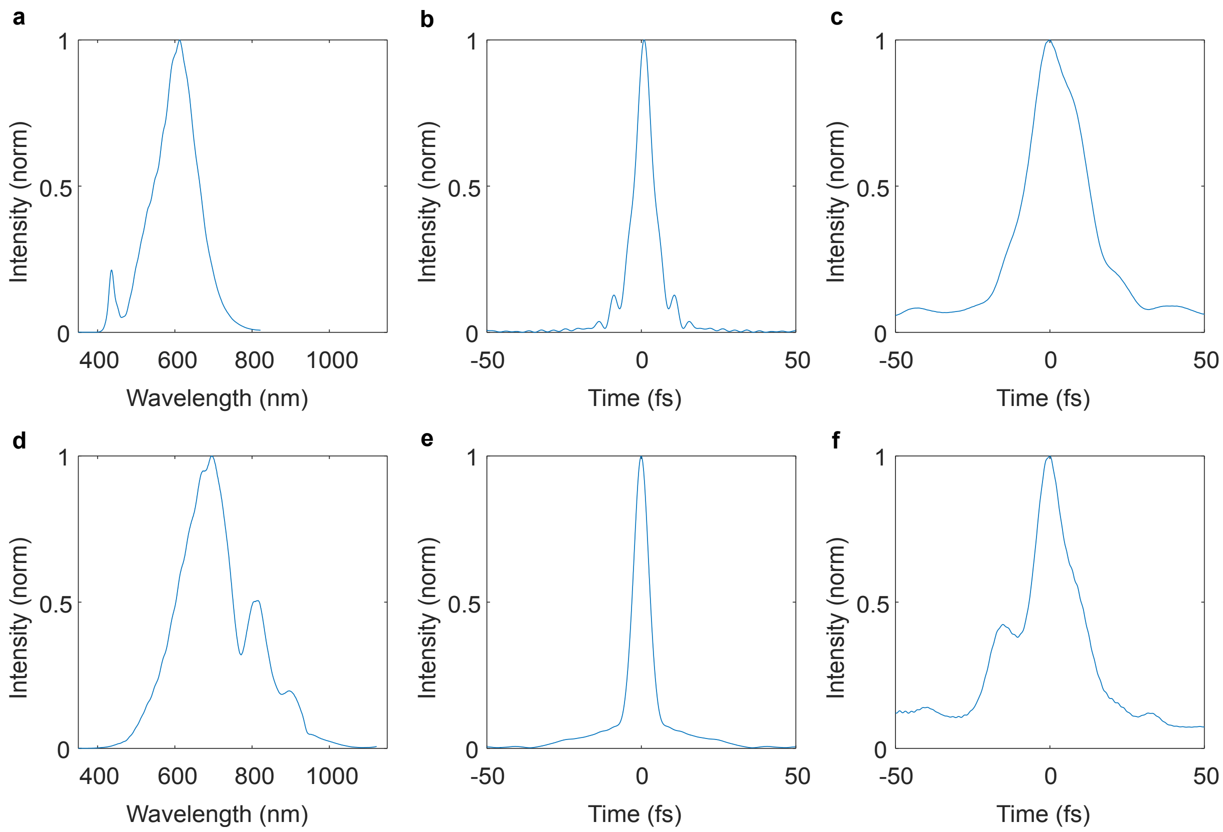

Supplementary Figure 5: **Spectra and resulting auto-correlation of the white light sources used in our experiments.**: **(a)** White LED source measured spectrum. **(b)** Absolute value of the field auto-correlation, calculated by a Fourier transform of the spectrum in (a). **(c)** Measured interference fringes envelope as detected on the camera using the LED source in our experiments through a diffusive barrier. **(d-f)** same as (a-c) for the halogen lamp used in the experiments of Fig. 5.

In addition to calculating the coherence time of each of the sources, we have compared the TOF resolution obtained with the two sources in our experiments. This was done by comparing the envelope of the interference fringes (as detected with a spectrogram analysis) obtained in the localization experiments with the two sources (Fig. 3 and Fig. 5) for a single source/object. Taking the FWHM of the envelope gives an estimate of the experimental TOF resolution of $\tau_{FWHM} \approx 21$fs for the LED source and $\tau_{FWHM} \approx 17$fs for the tungsten-halogen lamp (Supplementary Figure 5c,f). The TOF resolution in experiments is lower than the coherence-time of the sources due to the presence of the diffusive barrier, optical aberrations of the setup, and the effects of windowing of the spectrogram analysis. The experimental coherence length is given by $l_c = \tau_c \cdot c = 6.3\mu m$ for the LED source.

5.2. Fulfilling the spatial Nyquist sampling criteria

In order to be able to measure the interference fringes, the interference pattern has to be spatially Nyquist sampled, i.e. the camera pixel pitch Δx_{pixel} has to be smaller than half the spatial period of the white light fringes $\Lambda/2$. This period is dictated by the central wavelength of the light source, λ_0, and the geometry of the measurement system (Supplementary Figure 6): In our setup, the interference fringes are a result of interference between two apertures separated by a distance $D = 3.2/1.2$mm (the factor 1.2 is the reduced image of the slits on the barrier). The aperture mask is placed at the front focal plane of a lens ($f = 100$mm), and the camera is positioned in the back focal plane of the lens. In this geometry the spherical wave that is transmitted through each of the slits becomes a plane wave propagating at an angle $\theta = atan(D/2f)$ after passing through the lens. The low coherence interference fringes have a period which is $\Lambda = \lambda_0/2sin(\theta) \approx \frac{\lambda_0 f}{D}$. In our setup $D = 3.2/1.2$mm and $f = 100$mm were chosen such that $\Lambda \approx 22\mu\text{m}$, which is approximately three times larger than the camera pixel pitch $\Delta x_{\text{pixel}} = 6.5\mu\text{m}$, fulfilling the required Nyquist sampling criterion.

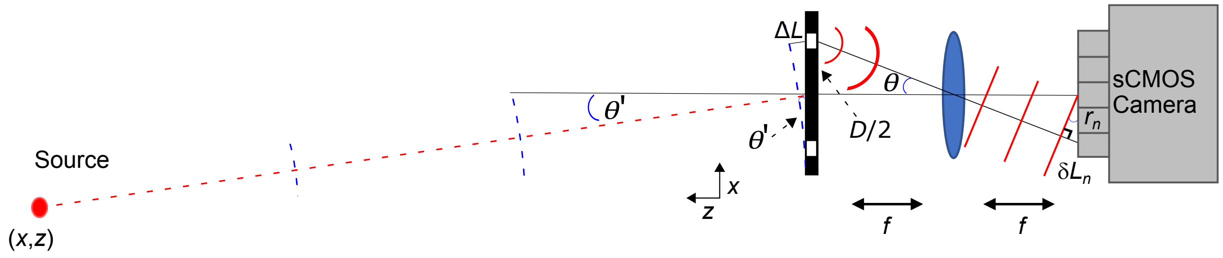

Supplementary Figure 6: **System geometry and path length difference δL_n to the different camera pixels, located at r_n.**

5.3. Camera pixel to TOF difference conversion

The calculation of the optical path length difference, δL (i.e. the time delay times the speed of light, $\delta L = c\Delta t$) for each pixel in the camera plane is depicted in Supplementary Figure 6. The position of the fringes on the camera, r_n, is directly converted to the TOF delay by considering that the relative path length difference from the on-axis camera pixel to the nth pixel is $\delta L_n = r_n sin(\theta)$, where $r_n = \Delta x_{\text{pixel}} \cdot n$ is the position of the nth pixel. Thus, the time delay corresponding to fringes measured at the nth pixel position is given by:

$$\Delta T_n = 2\delta L_n/c = 2r_n \cdot \sin(\theta)/c \approx \frac{r_n \cdot D}{c \cdot f} \tag{1}$$

In the last step we assumed $f \gg D, r_n$, as is the case in our experiments.

Substitution of our setup chosen parameters: $f = 100$mm, $D = 1.5/1.2$mm, and $r_n = n \cdot 6.5\mu$m, will give the time delay for the nth pixel:

$$\Delta T_n \approx n \cdot 0.3\text{fs} \tag{2}$$

resulting in a conversion of each camera pixel in our spatio-temporal maps to a TOF delay of 0.3fs, roughly a third of the central wavelength, as required for proper Nyquist sampling.

The number of time delays that can be sampled in a single camera image is limited by the number of pixels in a single camera row. In our experiments this number was limited to 2,160 pixels by the camera pixel count, and to 1400 pixels by the specific optics used, which limited the angular field-of-view of the speckles collected on the camera (the diameter of the speckled circle in Fig. 2b).

Supplementary Note 6:

Spatial localization resolution

In this section we provide an analytical derivation of the spatial localization resolution as derived from the TOF temporal resolution, and present experimental results quantifying the localization resolution in our experiments.

6.1. Theoretical estimate

Consider a distant object located at a distance z from the barrier, and at a transverse position x from the center of the measurement position. The light from this object arrives to the barrier at an angle of $\theta' = atan(x/z)$ measured in respect to the normal of the barrier (Supplementary Figure 6). The TOF difference in arrival time of the light from the object to the two measurement apertures having a separation D is:

$$\Delta t = \Delta L/c \approx D\sin(\theta')/c \tag{3}$$

This TOF difference is measured in our approach with a resolution that is given by the source coherence time: $\tau_{\mathrm{c}} = l_{\mathrm{c}}/c$, where l_{c} is the illumination coherence length. The angular localization resolution, $d\theta'$ can be estimated by equating the differential of the measured time delay, Δt, to the measurement temporal resolution τ_{c}:

$$\tau_{\mathrm{c}} = d(\Delta t) \approx D\cos(\theta')d\theta'/c \tag{4}$$

yielding:

$$d\theta' \approx l_{\mathrm{c}}/(D\cos(\theta')) \tag{5}$$

Substituting our experimental parameters: $l_{\mathrm{c}} = 6.3\mu\mathrm{m}$ (see Supplementary Note 5) and $D = 1.25\mathrm{mm}$ at $\theta' \approx 0$, we obtain $d\theta' \approx 5mrad$ for our experimental geometry.

The transverse (dx) and axial (dz) resolutions can be similarly derived by plugging into Supplementary Equation (3): $sin(\theta') \approx (x + x_{\mathrm{slits}})/z$, where x_{slits} is the center position of the moving slits, and taking the differential of Δt with respect to x and z separately, yielding:

$$d(\Delta t) \approx \frac{D}{c \cdot z} dx \tag{6}$$

and:

$$d(\Delta t) \approx \frac{D(x + x_{\text{slits}})}{c \cdot z^2} dz \tag{7}$$

Thus, the theoretically expected transverse and axial localization resolutions are given by:

$$dz \approx \frac{l_{\text{c}} \cdot z^2}{D(x + x_{\text{slits}})} \tag{8}$$

$$dx \approx l_{\text{c}} \cdot z/D \tag{9}$$

Substituting our experimental parameters of $l_{\text{c}} = 6.3\mu\text{m}$, $x = 0$, $x_{\text{slits}} = 10\text{mm}$, and $z = 80\text{cm}$ yields $dz \approx 32\text{cm}$, and $dx \approx 4\text{mm}$ for this distance.

The localization resolution is improved for larger apertures separation, D (i.e. a larger base-length). However, the apertures separation cannot be arbitrarily large when extended sources are concerned. In order to obtain high contrast fringes for an extended source, the apertures separation must be smaller than the coherence size of the source at the barrier, r_{coh}. According to the Van-Cittert Zernike theorem, $r_{\text{coh}} \approx z\lambda/D_{\text{obj}}$, where D_{obj} is the object's diameter. The largest apertures separation providing high contrast fringes is thus of the order of the coherence size: $D \approx r_{\text{coh}}$. This will yield an angular resolution from a single camera shot of:

$$d\theta' \approx l_{\text{c}}/(r_{\text{coh}} \cdot \cos(\theta')) \approx l_{\text{c}} \Delta\theta'_{\text{obj}}/(\lambda \cdot \cos(\theta')) \tag{10}$$

where $\Delta\theta'_{\text{obj}} = D_{\text{obj}}/z$ is the angular size of the hidden source.

For broadband white-light sources the coherence length, l_{c} is of the order of the wavelength, λ . Thus, the single-shot localization resolution for extended sources is comparable to the object angular size.

The final localization accuracy is considerably better than the single shot localization resolution, since it is obtained from multiple single-shot TOF measurements, as is studied experimentally in Supplementary Note 7.

6.2. Experimental localization resolution verification

To verify the theoretical prediction of the localization resolution we have performed a set of experiments with two white-light sources placed at various small axial and transverse separations from each other. The experimental results of the transverse localization resolution are presented in Supplementary Figure 7. Supplementary Figure 7a, displays the measured spatio-temporal (x-t) TOF trace for two sources positioned at $z = 60\text{cm}$, separated by $\Delta x = 3\text{mm}$, as measured with our optical setup and processed by spectrogram analysis (see Supplementary Note 3). At this distance, the theoretical transverse localization resolution (Supplementary Equation 9) is expected to be $dx \approx 3\text{mm}$. Indeed, the two sources that are separated by this transverse spacing

are resolved, but with a small contrast, as is also visible in Supplementary Figure 7b, where the cross-section of a single row of the trace of Supplementary Figure 7a is plotted.

We have repeated this experiment for different transverse separations $\Delta x = 3 - 12$mm, for two longer distances from the barrier: $z = 110$cm and $z = 130$cm. The results of this study are presented in Supplementary Figure 7c,d. As expected from the theoretical estimation, sources separated by a transverse spacing of $\Delta x = 3$mm are no longer resolved at the longer distances, and the individual sources are distinguished only for separations of $\Delta x = 6$mm for $z = 110$cm (Supplementary Figure 7c), and $\Delta x = 7$mm for $z = 130$cm (Supplementary Figure 7d), with good accordance with the theoretical estimations of Supplementary Equations (9).

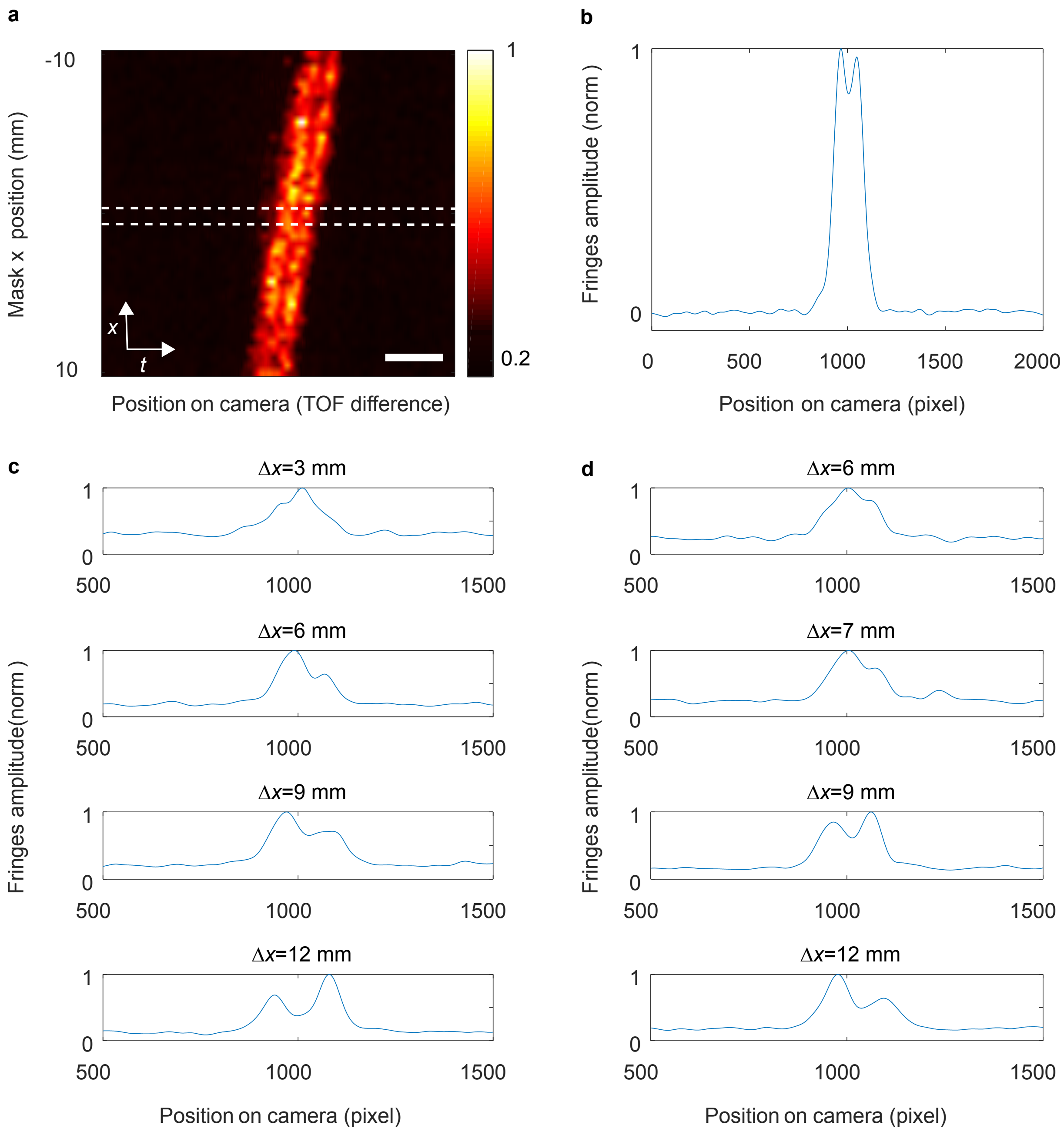

Supplementary Figure 7: **Transverse localization resolution, experimental results.** **(a)** Measured spatio-temporal traces for two light sources separated transversely by $\Delta x = 3$mm at a distance of $z = 60$cm behind the diffuser. **(b)** Envelope of the interference fringes for a single mask position taken from the cross-section marked by the dashed box in (a) . **(c)** Same as (b) for two sources with various transverse separations, located at a depth of $z = 110$cm from the barrier. **(d)** Same as (c) for two sources at $z = 130$cm. Scale bar: 100fs.

The experimental results for axial localization resolution measured with our optical setup are summarized in Supplementary Figure 8. In each of these four experiments, two sources were present in the scene at the same transverse position, but at different depths. In all of these experiments one source is located at a fixed distance $z_1 = 110$cm behind the diffuser, and the second source is located at a distance of $z_2 = 30$cm, 45cm, 60cm, 80cm from the barrier respectively, with no transverse translation. The four traces show the envelope of the interference fringes position on the camera as a function of the mask's position.

For the sources separated by $\Delta z = 50$cm or larger (Supplementary Figure 8a-c) the two sources are clearly resolved at the top and bottom of the spatiotemporal traces, i.e. when the mask is shifted by the largest transverse distance, as expected from the theoretical prediction of Supplementary Equation 8. However, the sources separated by $\Delta z = 30$cm (Supplementary Figure 8d) are no longer resolved, in agreement with the theoretical prediction of Supplementary Equation (8). Verifying our theoretical analysis for the technique localization resolution.

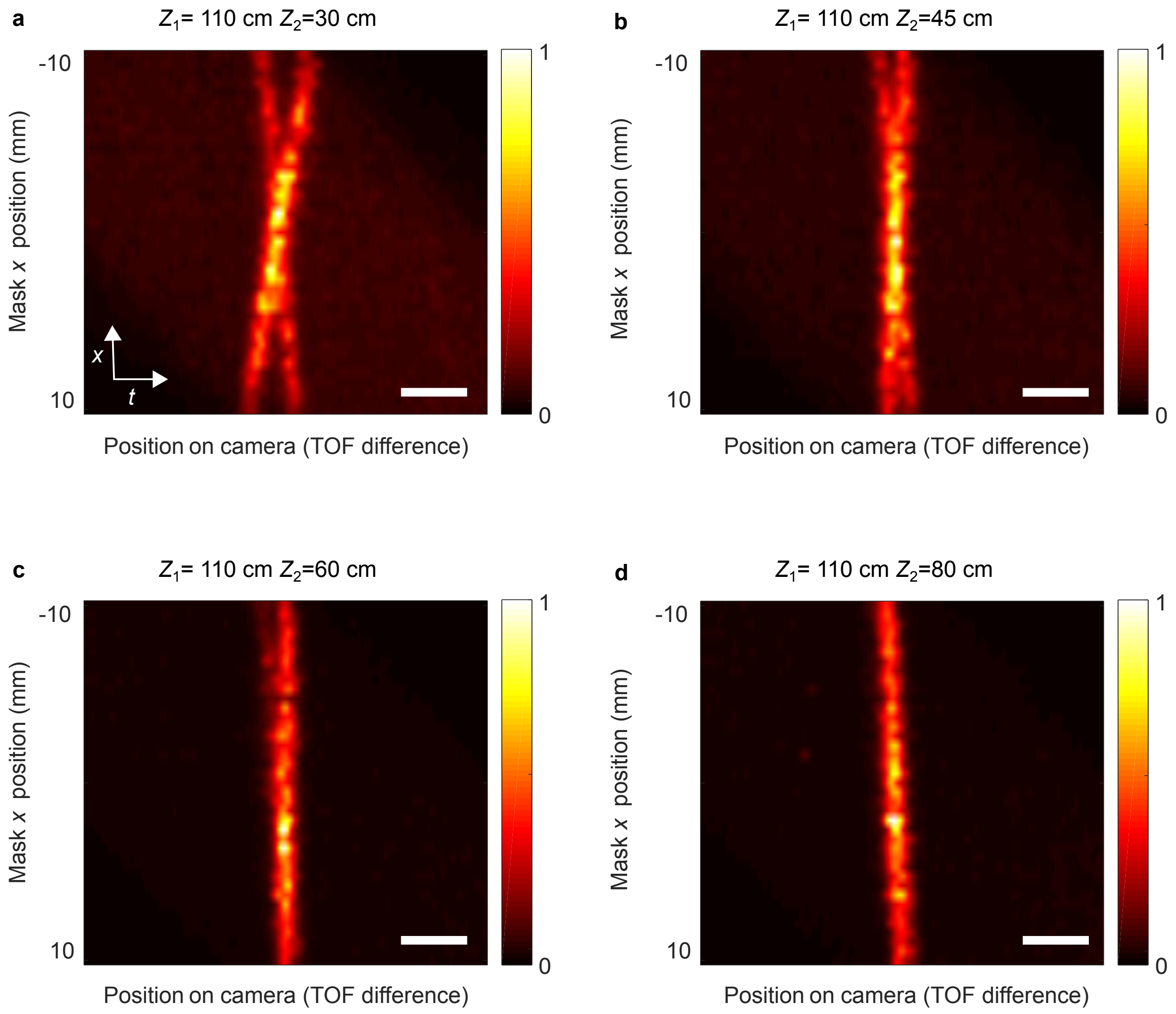

Supplementary Figure 8: **Axial localization resolution, experimental results. (a-d)** Envelope of the interference fringes position on the camera as a function of the position of the mask, for two light sources positioned at varying axial (depth) separations, $\Delta z = 30 - 80$cm behind the diffuser, at the same transverse position. The depths of the two sources are marked by Z_1 and Z_2 above each trace. Scale bars: 100fs.

Supplementary Note 7:

Localization accuracy

As mentioned in the main text, the localization accuracy is significantly better than the localization resolution, since it is obtained from multiple, high-SNR measurements. In order to study

the transverse and axial localization accuracy in our experiments as a function of the number of TOF measurements taken (number of different mask's positions), we have acquired TOF data for a single source located at various transverse positions at a distance of $z = 57$cm behind the diffusive barrier . For each source position, we have localized the source from a different number of TOF measurements ranging from 2 to 42. From each TOF measurement a plot of the corresponding hyperbola was back-projected (Supplementary Figure 9a). The point of intersection of the back-projection hyperbolas was found by convoluting the hyperbolas with a spatial blurring kernel of size 40 by 40 pixels in the reconstruction grid (0.4mm along the vertical axis (x) and 4.8cm along the horizontal axis (z) of Supplementary Figure 9a) and marking the peak of the resulting trace as the estimated position of the source. These positions together with the back-projected hyperbolas are presented in Supplementary Figure 9a: The reconstructed positions are marked by blue 'x', and the real positions of the source are marked by white 'x'.

The localization accuracy was estimated by calculating the standard deviation of the estimated depths (Δz) and the transverse (Δx) positions, from the real positions of the sources. The results for the localization accuracy in depth and transverse position as a function of the number of TOF measurements (number of back-projected hyperbolas) are presented in Supplementary Figure 9b,c, respectively. As expected, the localization accuracy is improved when more TOF measurements are used. At this depth ($z = 57$cm) the transverse localization accuracy with our experimental parameters reaches 0.2mm, and the depth resolution reaches $\Delta z = 1$cm. The transverse resolution is two orders of magnitude better than the axial resolution in our experiments, as expected from the localization resolution (see Supplementary Note 6).

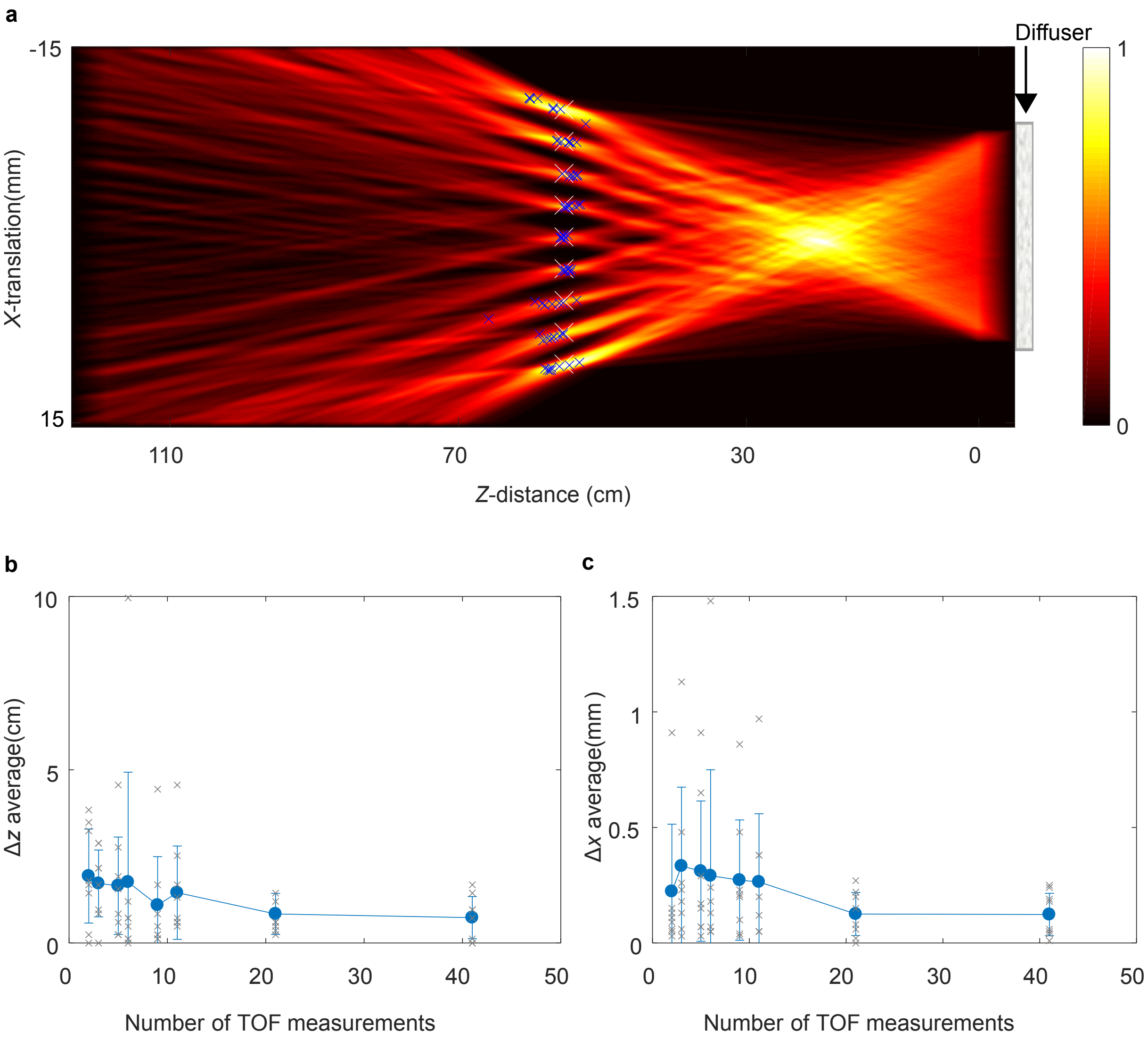

Supplementary Figure 9: **Localization accuracy of a single hidden source as a function of the number of TOF measurements (mask positions).** **(a)** Plot of back-projected hyperbolas from all TOF measurements for a single source placed at several transverse positions at a distance z=57cm behind the diffuser. The intersections of the hyperbolas reveal the different reconstructed positions (marked by blue 'x'), using a different number of TOF measurements. The real positions are marked by white 'x'. **(b)** Axial localization accuracy as a function of the number of TOF measurements, as calculated from the standard-deviation of the reconstructed positions in (a) (blue dot), the different realizations are marked by gray 'x'. **(c)** Same as (b) for the transverse localization accuracy. Error bars, standard deviations of nine different realizations.

Supplementary Note 8:

Effects of ambient light and exposure time

In this section we experimentally study the effects of ambient lighting, background reflections, and exposure time, and the measured fringe contrast. Similar to OCT, beyond the absolute signal level, one of the main challenges of our passive TOF technique is the signal to background ratio, or the fringe contrast. The experimental results presented in Figures 2-5 were performed under dark conditions. In order to study the practical effects of ambient light illumination and white reflective background, we have performed a set of measurements whose results are summarized in Supplementary Figure 10. Supplementary Figure 10 shows an analysis of the fringes envelope SNR, under different illumination conditions and exposure times. The SNR was quantified by taking the amplitude ratio between the peak of the fringes envelope and the standard deviation of the background signal in a single camera shot (a single row in Supplementary Figure 10a-c). Three scenarios were considered: the first is a dark conditions scenario (Supplementary Figure 10a,d), the second is a scene with ambient room light (Supplementary Figure 10b,e), and the third is under ambient room light in addition to a white painted cardboard placed 20cm behind the hidden LED source (Supplementary Figure 10c,f). The results of these experiments are summarized in Supplementary Figure 10g. As expected, the presence of ambient light, a reflective background, and shorter exposure times, all lower the fringe contrast as quantified by the SNR. However, localization of small white-light LED sources is possible even under ambient light conditions.

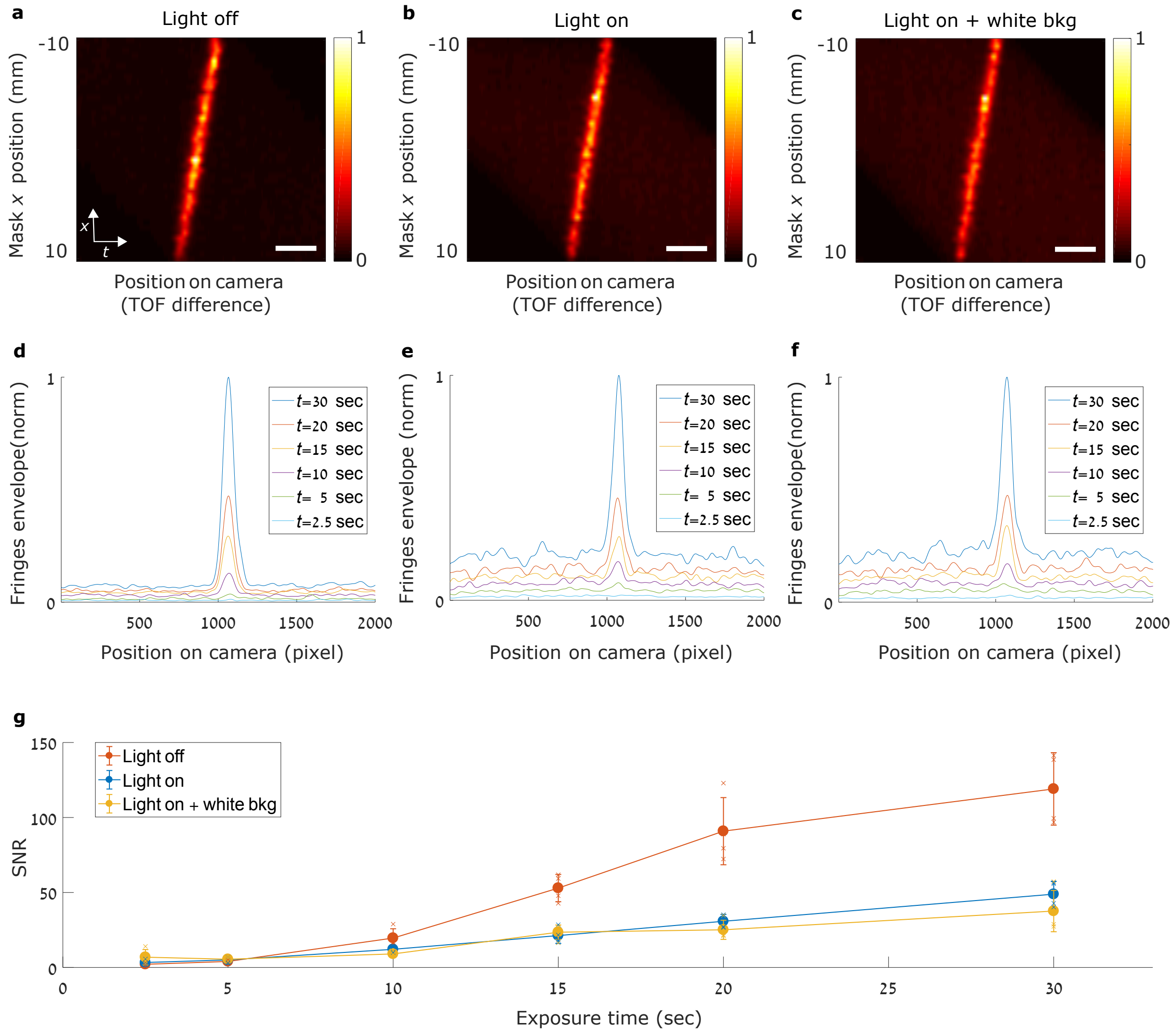

Supplementary Figure 10: **Effect of ambient illumination, background reflection, and exposure time on experimentally-measured fringe contrast.** **(a-c)** Examples spatio-temporal traces for a single source in three different scenarios: **(a)** a dark room, **(b)** with ambient room light, and **(c)** with ambient room-light and a white-painted cardboard located 20cm behind the LED light source. Exposure time per row is 30sec. **(d-f)** cross-sections of a single row in (a-c), for different exposure times. **(g)** Calculated signal to noise ratio (SNR) of the fringes as a function of the exposure time for the measurements shown in (d-f) (orange, blue and yellow respectively), the different realizations are marked by 'x'. Error bars, standard deviations of four different realizations. Scale bars: 100fs.

Supplementary Note 9:

Characterization of white-painted wall reflection

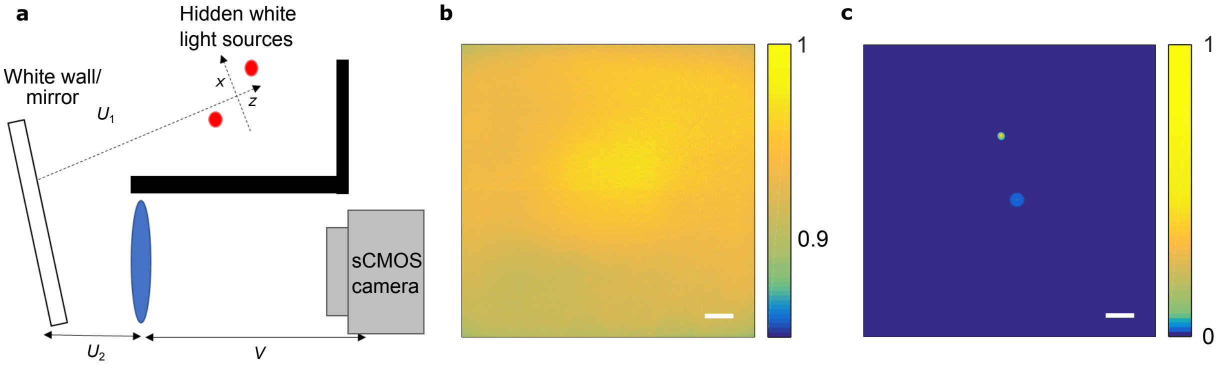

Supplementary Figure 11: **Verification for no specular reflection information by conventional imaging:(a)** Conventional imaging setup: the two light sources of Fig.4 are placed around the corner. A single lens with a focal length f images the plane between the two points (located at a distance $U_1 + U_2$ from the lens) on the camera, located at a distance V from the lens, such that $1/(U_1 + U_2) + 1/V = 1/f$. **(b)** The image obtained from light reflected by the white diffusive wall is featureless, showing no specular reflection. **(c)** The image obtained with a mirror replacing the wall, showing clearly the two light sources. Scale bars: 1cm.

In the around the corner localization experiments of Fig. 4, the light was collected at a reflection angle approximately equal to the angle of incidence of the light from the object. This was done in order to maximize the light collection efficiency, and speckle contrast. The latter is maximized as the speckle spectral decorrelation width is maximized, which occurs in this angle [5]. In order to verify that there is no significant specular reflection at this angle that can reveal the position of the light sources with conventional imaging, we have performed conventional imaging with the light reflected from the wall, and compared to the case when the wall was replaced by a mirror.

The results of these measurements are displayed in Supplementary Figure 11. The setup (Supplementary Figure 11a) is a simple single lens imaging setup, which images the two hidden objects of Fig. 4a, on a camera. The light source that is closer to the wall is located at a distance $U_1 + U_2 = 56 + 14 = 70$cm from the lens ($f = 8$cm), and an sCMOS camera is positioned at a distance $V = 9$cm behind the lens, such that $(U_1 + U_2)^{-1} + (V)^{-1} = f^{-1}$.

Supplementary Figure 11b shows the image recorded using the light reflected from the white painted wall, showing no information on the hidden objects positions. In contrast, replacing the wall with a mirror wall clearly reveals the positions of the two light sources (Supplementary Figure 11c).

Supplementary Note 10:

Wall roughness effects on TOF measurement

Supplementary Figure 12a,b compares the TOF traces for localization through a scattering diffuser (Supplementary Figure 12a) and localization using light scattered off a white painted wall (Supplementary Figure 12b). It can be observed that in the white-painted wall case, there are considerable variations of the TOF trace from a linear curve. These variations are due to the roughness of the wall. As mentioned in the main text, these TOF variations contain information on the barrier and can provide a measure of the barrier roughness.

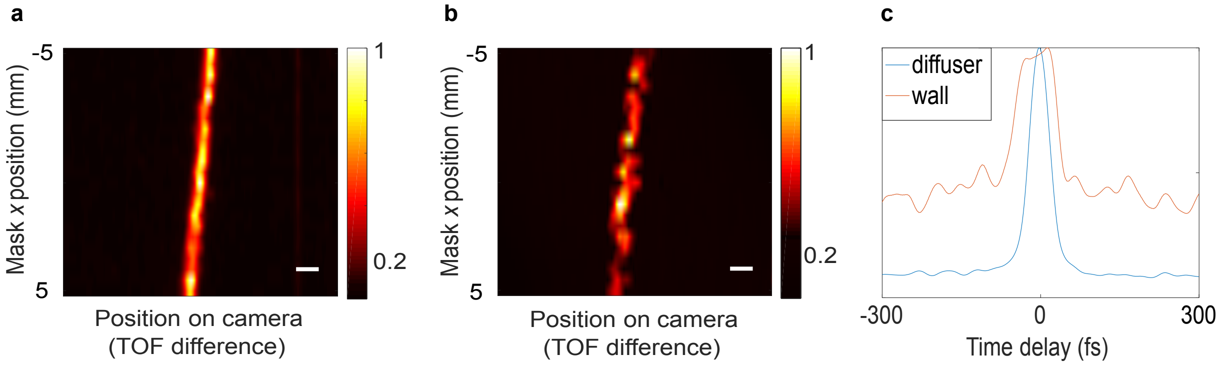

Supplementary Figure 12: **Comparison of TOF traces obtained through a diffuser and from white-painted surface scattering. (a-b)** Traces of the interference fringes envelope as a function of the mask position for localization through a diffuser (a) and for light reflected from white-painted surface (b). **(c)** Cross sections of a single row in (a)(blue) and (b) (orange). Scale bars: 100fs.

In addition to the variations in the positions of the peak of the different TOF traces, the temporal width of a single TOF trace taken with the white-painted wall is broader than the one taken with the diffuser. The broader response of the wall is a result of the wall being an effective thick scatterer, producing a larger spread in optical paths of the back-scattered light, as is analyzed theoretically in Supplementary Note 11, below. The width of the single TOF path can be used to study the scattering properties of the barrier, such as its transport mean free path [6].

Supplementary Note 11:

Influence of a thick barrier on the temporal cross-correlation

In this section we theoretically analyze the potential distortions induced by multiple-scattering in a thick barrier on the measured temporal cross-correlation. Consider two fields $E_1(t)$ and $E_2(t)$, arriving at the diffusive barrier at two different points, as depicted in Supplementary Figure 13. Each of the fields exiting the barrier $E_{\mathrm{i,m}}$, which are measured by our system, are given by the convolution of the entering field with the impulse response function of the barrier, $h_i(t)$, for the specific input-output positions r_{i}, such that:

$$E_{1,\mathrm{m}}(t) = E_1(t) * h_1(t) \tag{11}$$

$$E_{2,\mathrm{m}}(t) = E_2(t) * h_2(t) \tag{12}$$

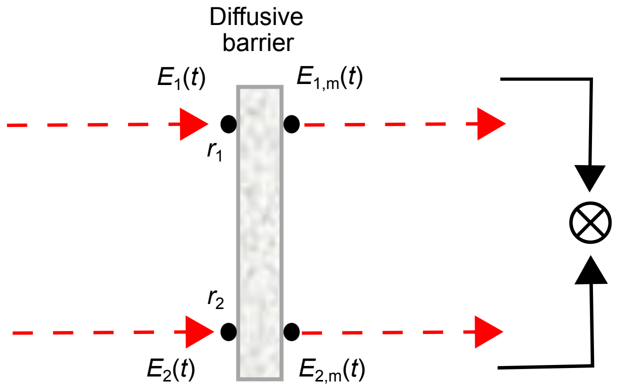

Supplementary Figure 13: **The influence of the impulse response of the barrier**

The cross-correlation of the measured fields exiting the barrier is thus given by:

$$E_{1,\mathrm{m}} \otimes E_{2,\mathrm{m}}(t) = [E_1(t) * h_1(t)] \otimes [E_2(t) * h_2(t)] \tag{13}$$

Since all the fields and impulse responses are real functions, the cross-correlation of the convolutions is equal to the convolution of the autocorrelations:

$$E_{1,\mathrm{m}} \otimes E_2(t) = [E_1(t) \otimes E_2(t)] * [h_1(t) \otimes h_2(t)] \tag{14}$$

For a sufficiently thin barrier the impulse responses, $h_i(t)$, can be approximated as delta functions, thus providing a good estimate for the arriving fields cross-correlations, as required:

$$E_{1,\mathrm{m}} \otimes E_{2,\mathrm{m}}(t) \approx E_1(t) \otimes E_2(t) \tag{15}$$

In the case of thick multiply scattering barriers, the measured cross-correlation will be the convolution of the desired cross-correlation with the cross-correlation of the impulse responses, which is a function that is limited in time to twice of the Thouless time (dwell time) of the light in the medium. Thus, a thick scattering barrier will induce distortions and smearing that are given by the path delay spread of the light in the medium. While effectively lowering the temporal resolution, the temporal cross-correlation approach is effective also through effectively thick barriers, as we prove experimentally in the results presented in Fig. 4.

Supplementary References

[1] P.G. Tuthill, J.D. Monnier, W.C. Danchi, E.H. Wishnow, C.A. Haniff, Michelson interferometry with the Keck I telescope. *Publications of the Astronomical Society of the Pacific*, 112, 555-565 (2000).

[2] A. Velten, T. Willwacher, O. Gupta, A. Veeraraghavan, M. Bawendi, G. Moungi, R. Raskar, Recovering three-dimensional shape around a corner using ultrafast time-of-flight imaging. *Nature communications*, 3, 745 (2012).

[3] G. Gariepy, F. Tonolini, R. Henderson, J. Leach, D. Faccio, Detection and tracking of moving objects hidden from view. *Nature Photonics*, 10, 23–26 (2016).

[4] M. OToole, D. Lindell, G. Wetzstein, Confocal non-line-of-sight imaging based on the light-cone transform. *Nature*, 555, 338-341 (2018).

[5] E. Small, O. Katz, Y. Guan, Y. Silberberg, Spectral control of broadband light through random media by wavefront shaping. *Optics Letters*, 37, 3429–3431 (2012).

[6] A. Badon, G. Lerosey, A.C. Boccara, M. Fink, A. Aubry, Retrieving time-dependent Greens functions in optics with low-coherence interferometry. *Physical Review Letters*, 114, 023901 (2015).
